# Supplementary figures and images for: The impact of genetic background and sex on the phenotype of IL-23 induced murine spondyloarthritis
Source: PLoS One. 2021 May 13;16(5):e0247149. doi: 10.1371/journal.pone.0247149 (PMC8118278; doi:10.1371/journal.pone.0247149)

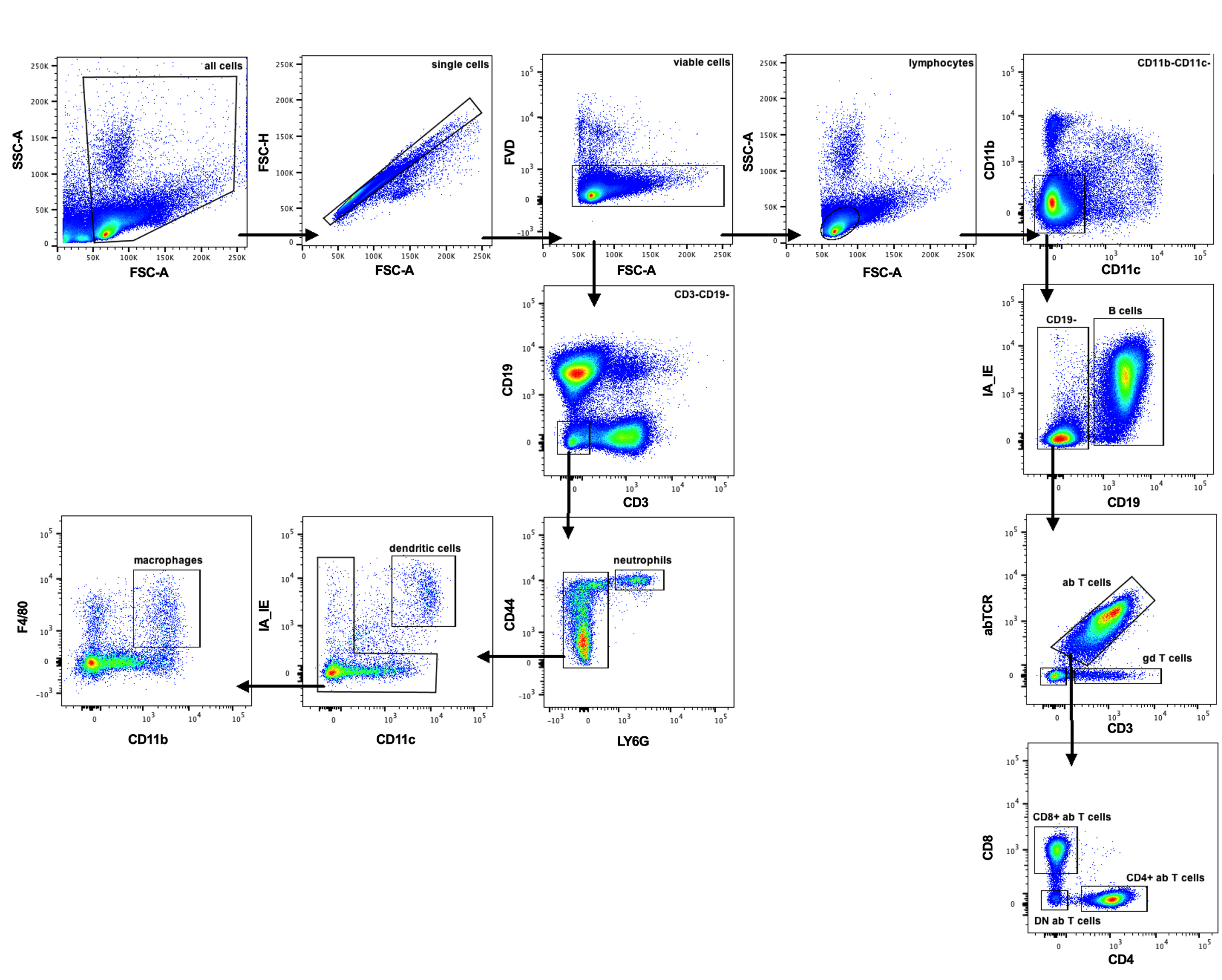

Supplement: S1 Fig — Cell analysis was performed on BD LSRFortessa. Gating was performed using FlowJo software. (TIF) [file pone.0247149.s001.tif]

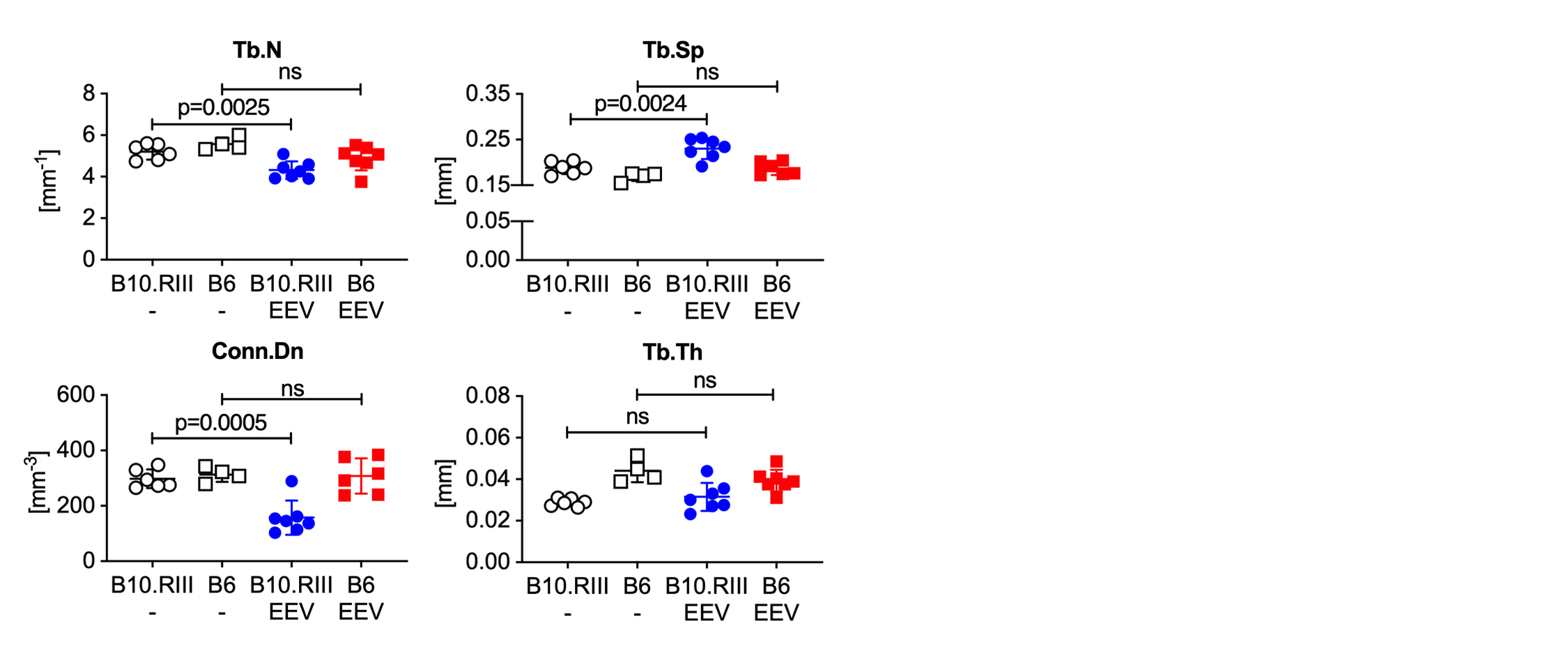

Supplement: S2 Fig — μCT trabecular bone parameters from femurs of control and IL-23 EEV injected B10.RIII and B6 14 days after IL-23 EEV injection (n = 4–7 mice/group). Trabecular number (Tb. N), trabecular spacing (Tb. Sp), connective density (Conn.Dn), and trabecular thickness (Tb.Th). P values were determined by unpaired t-test. (TIF) [file pone.0247149.s002.tif]

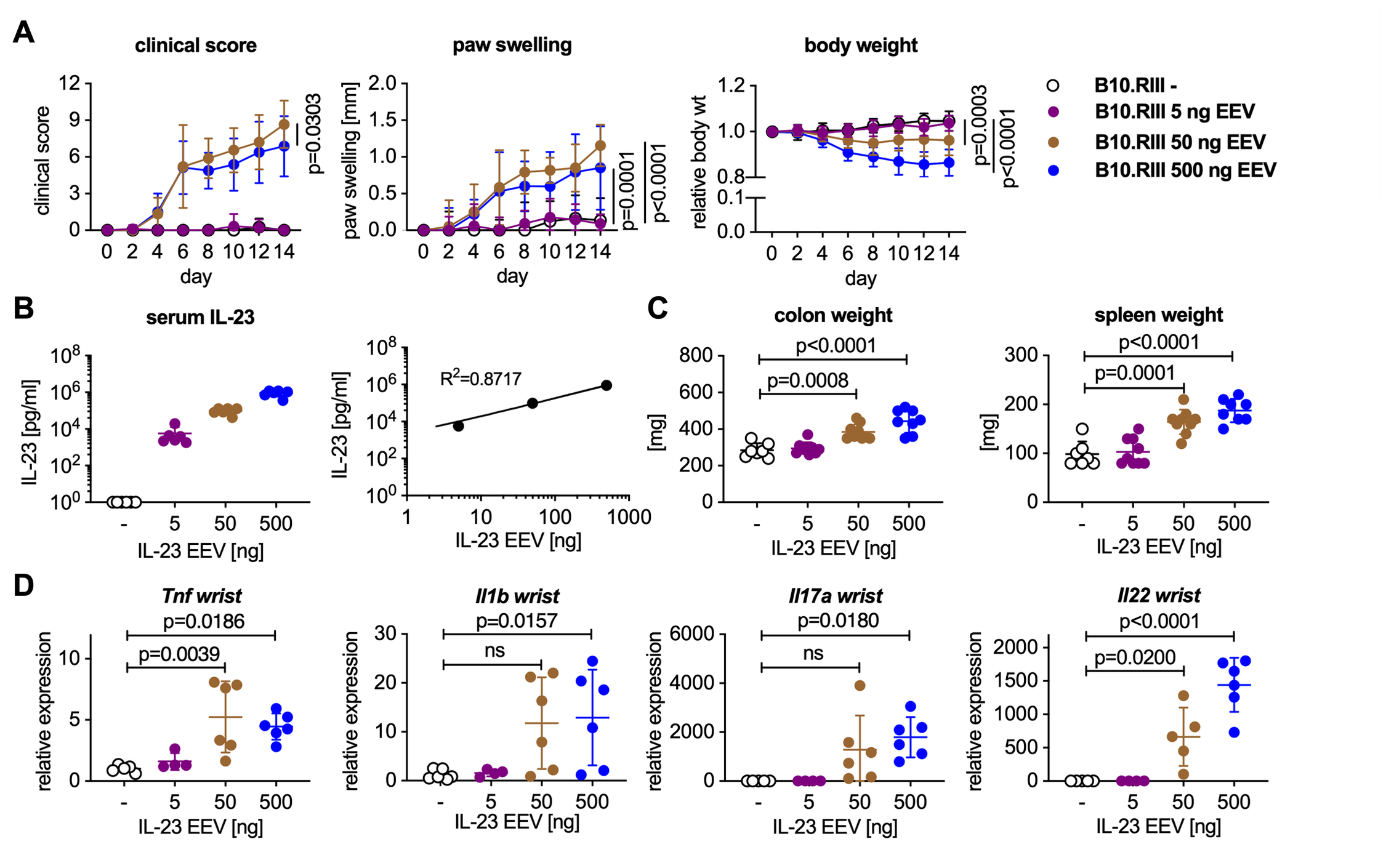

Supplement: S3 Fig — 8–12 week-old male mice received 5, 50, or 500 ng IL-23 EEV on day 0 via hydrodynamic tail vein injection; control mice received no injection (n = 7–8 mice/group). (A) Clinical score (mean ± SD), paw swelling (mean ± SD), body weight (mean ± SD). (B) Serum IL-23 on day 14 was determined by ELISA. (C) Spleen weight, colon weight. Dotted lines represent the mean weights for control mice. (D) Gene expression analysis in the wrists on day 14. qPCR data for individual samples were first normalized by Hprt expression and then divided by the mean of the uninjected control group. (TIF) [file pone.0247149.s003.tif]
